# Proteotoxic stress response in atherosclerotic cardiovascular disease: Emerging role of heat shock factor 1

**DOI:** 10.3389/fcvm.2023.1155444

**Published:** 2023-04-03

**Authors:** Shruti Ghai, Alex Young, Kuo-Hui Su

**Affiliations:** Department of Cell and Cancer Biology, College of Medicine and Life Sciences, The University of Toledo, Toledo, OH, United States

**Keywords:** heat shock proteins, heat shock factor 1, foam cells, atherosclerosis, lipid metabolism

## Abstract

Atherosclerosis is a major risk factor for cardiovascular diseases. Hypercholesterolemia has been both clinically and experimentally linked to cardiovascular disease and is involved in the initiation of atherosclerosis. Heat shock factor 1 (HSF1) is involved in the control of atherosclerosis. HSF1 is a critical transcriptional factor of the proteotoxic stress response that regulates the production of heat shock proteins (HSPs) and other important activities such as lipid metabolism. Recently, HSF1 is reported to directly interact with and inhibit AMP-activated protein kinase (AMPK) to promote lipogenesis and cholesterol synthesis. This review highlights roles of HSF1 and HSPs in critical metabolic pathways of atherosclerosis, including lipogenesis and proteome homeostasis.

## Introduction

Atherosclerosis is the leading cause of cardiovascular disease, it is a pathological condition that leads to coronary heart disease, stroke, and peripheral arterial diseases by narrowing the lining of the coronary arteries, cerebral arteries, iliac arteries, femoral arteries, and aorta (1–3). Atherosclerosis typically develops at branch points and curvatures in large arteries where disturbed blood flow creates a pro-atherogenic environment that leads to macrophage accumulation and foam cell formation with the deposition of primarily cholesterol and its esters in the intimal macrophage at early stage of the disease (4). Increased lipid deposition at certain places and the subsequent formation of a cap of smooth muscle and fibrous tissue around a core of lipid and necrotic debris are both seen in atherosclerotic patients (2). These alterations lead to the development of raised lesions termed fibrous plaques, which extend into the lumen and start to disrupt blood flow.

Maintaining a healthy level of cholesterol in the body is essential to prevent atherosclerosis. Homeostasis of cholesterol includes cholesterol absorption, trafficking, and efflux (5). Macrophages are responsible for the absorption of oxidized low-density lipoprotein (oxLDL) and the accumulation of the lipid in the cells (4). If cholesterol is accumulated excessively in macrophages and smooth muscle cells (SMCs), it would contribute to the formation of foam cells and cause atherosclerosis (3). Understanding the modulation of intracellular lipid homeostasis is important to comprehend atherosclerosis.

The intracellular protein homeostasis is required for lipid homeostasis (6, 7). The proteotoxic stress response (PSR) is important to keep the intracellular protein homeostasis stable and has been shown to play an important role in the development of atherosclerosis. Heat shock factor 1 (HSF1) is a transcriptional factor for the expression of heat shock proteins (HSPs) to maintain proteome homeostasis, it also participates in the regulation of the intracellular lipid homeostasis (8). However, roles that HSF1 and HSPs play in the development of atherosclerosis are not completely known. Importantly, HSPs either promote or inhibit atherosclerosis at various stages of atherosclerosis development (9–13). A comprehensive understanding of the relationship between HSF1/HSPs and atherosclerosis is therefore essential for the development of effective treatment approaches or the identification of clinically useful markers for atherosclerosis. This review will focus on discussing influences that HSF1 and HSPs have on atherosclerosis and focus on macrophage foam cells due to space limitation.

## Etiology of atherosclerosis

Atherosclerosis is caused by a number of physiological and pathological conditions, including chronic inflammation, dysregulation of lipid metabolism, oxidative stress, and incorrect immunological responses (3, 14, 15). Despite advancements in the study of atherosclerosis, the field lacks a systemic understanding of mechanisms of the disease, particularly in molecular regulators with significant pathophysiological commonalities between intracellular proteome homeostasis and atherosclerosis. Growing evidence supports the concept that aberrations in the metabolic programming contribute to the development of atherosclerosis (16, 17). Chronic inflammation induced by metabolic perturbance is also associated with disordered proteomic stability in atherosclerosis (18). Therefore, investigating how cellular metabolism responds to the PSR in cardiovascular disease has immense potential to answer these mechanistic questions and suggest new areas for further basic and translation investigations.

The formation of macrophage foam cells is a critical step in the pathogenesis of atherosclerosis. Early in the development of atherosclerosis, the endothelial dysfunction leads to macrophage cell infiltration and macrophage-derived foam cells proliferate and aggregate in the subendothelial space of a damaged artery (1–4). The processing and metabolism of cholesterol inside macrophages, which involve uptake modified lipoproteins, such as oxLDL, esterification, and outflow that culminates in cholesterol equilibrium, are among the most recognized crucial stages in the creation of foam cells. Dysregulations of the cholesterol equilibrium in macrophages lead to the accumulation of intracellular lipid droplets and the formation of characteristic foam-like structures (19, 20). Later stages of atherosclerosis are characterized by the presence of other cell types, including SMCs and SMC-derived cells (19, 21). SMCs can differentiate into foam cells themselves. SMCs can also contribute to the progression of atherosclerosis by phenotypic switching to a more synthetic state, leading to the formation of a fibrous cap that is prone to rupture (21–24). Several key molecular pathways and transcription factors that regulate SMC phenotypes and foam cell formation, including KLF4 that may be a potential therapeutic target for atherosclerosis, have been identified (25, 26). During the development of the atherosclerotic plaque, there is also a significant infiltration of other cell types that contributes to the progression of atherosclerosis, such as T lymphocytes, dendritic cells, and mast cells, into the intima (1, 15, 27, 28).

## Proteotoxic stress response (PSR)

Cellular proteome homeostasis (proteostasis) refers to the processes that mediate protein synthesis, folding, trafficking, complex assembly, and destruction inside cells (29). Proteotoxic stressors, such as heat, heavy metals, UV radiation, and hypoxia, perturb proteostasis and cause cellular protein damages or conformational changes, which lead to protein misfolding and can trigger diseases (30, 31). For example, heat stress dramatically alters cell physiology and might cause cell death, the outcome depends on the intensity of the stress (32). These proteotoxic stressors also trigger the PSR, which involves well-characterized cytoprotective mechanisms that produce HSPs (33). HSPs are molecular chaperones. The main purpose of molecular chaperons is to protect the proteome from damaging protein misfoldings and to reduce protein aggregation by regulating protein folding, trafficking, assembly, ubiquination, and proteasomal degradation (33). The PSR is critical for the survival of cells under proteotoxic stress and contributes to the pathophysiology of common human diseases. As a cytoprotective chaperon mechanism, the PSR is induced by diverse environmental stressors and results in several transcriptional changes (34). PSR can trigger HSF1, a transcription factor, to regulate the expression of HSPs (34).

HSF1 and HSPs confer protection against cardiovascular diseases, including atrial fibrillation, cardiac hypertrophy, and cardiomyopathy (35–37). HSPs have been shown to play crucial roles in atherosclerosis but they can both positively and negatively affect the development of atherosclerosis (9, 38). HSF1 has also been linked to atherosclerosis-related cardiovascular disorders, and evidence is mounting that HSF1 plays a crucial role in these conditions (39). PSR, therefore, is now increasingly appreciated to be a key regulator of atherosclerosis but its role in mediating cardiovascular complications of atherosclerosis remains to be further elucidated. Below, we will discuss recent results connecting HSPs (mainly HSP72 and HSP27 in this article) and the perspective role of HSF1 to atherosclerotic cardiovascular disease.

## HSPs in atherosclerosis

**Figure 1:**
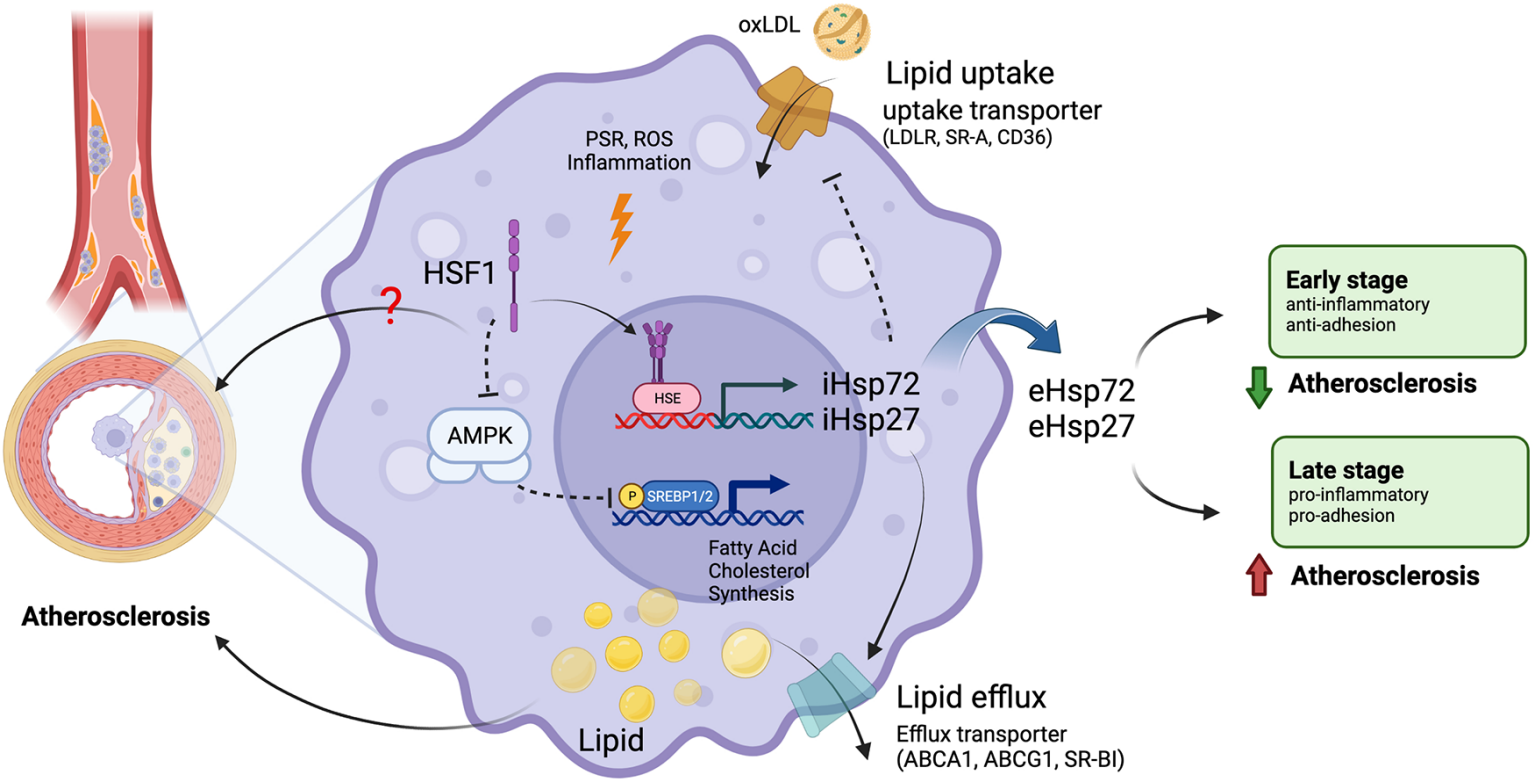
Roles of HSF1 in atherosclerosis. Macrophages uptake oxLDL through lipid uptake transporters and accumulate lipids intracellularly. Macrophages remove excessive intracellular lipids through lipid efflux transporters. Unbalanced lipid homeostasis leads macrophages to form foam cells, leading to the formation of atherosclerosis plaques. Chronic inflammation, PSR, and ROS induce the transcriptional function of HSF1 to express HSPs, such as HSP27 and HSP72. Intercellular HSPs (iHSPs) promote cholesterol efflux and inhibit lipid uptake. Secreted extracellular HSPs (eHSPs) play dual roles in atherosclerosis. HSPs inhibit atherogenesis during the early stage of atherosclerosis but promote atherosclerosis during the late stage of atherosclerosis. HSF1 non-transcriptionally promotes lipogenesis and cholesterol synthesis *via* SREBP1/2 by interacting with and inhibiting AMPK. ABCA1, ATP binding cassette subfamily A member 1; ABCG1, ATP binding cassette subfamily G member 1; AMPK, AMP-activated protein kinase; CD36, cluster of differentiation 36; eHSPs, extracellular heat shock proteins; HSE, heat shock element; HSF1, heat shock factor 1; HSPs, heat shock proteins; iHSPs, intracellular heat shock proteins; LDLR, low-density lipoprotein receptor; oxLDL, oxidized low-density lipoprotein; p, phosphorylation; PSR, proteotoxic stress response; ROS, reactive oxygen species; SR-A, scavenger receptor type A; SR-BI, scavenger receptor class B type I; SREBP1/2, sterol regulatory element binding protein 1/2. Images were created with BioRender.com.

HSPs, including HSP27, HSP72, HSP90, and HSP100, safeguard cells against stress, mainly by serving as molecular chaperones to repair denatured proteins by re-forming their native three-dimensional structures in order to maintain the proteostasis (40). Ample evidence supports that HSPs are involved in atherosclerosis (Figure 1) (9–11). However, it is still unclear how expressions of HSPs affect the development of atherosclerosis.

Foam cell formation is a hallmark of early atherosclerosis, mainly from macrophage at the earlier stage and from SMCs at late stage (27). Foam cell formation from macrophages is through lipid absorption, efflux, and cholesterol esterification. Inadequate cholesterol efflux may result in the accumulation of lipids and cholesterol esters in macrophages (41). This critical step of cholesterol efflux is carried out by ATP binding cassette subfamily A member 1 (ABCA1), ABCG1, and scavenger receptor (SR)-BI. ABCA1 and ABCG1 transport lipid-deficient Apolipoprotein (apo) A-I free cholesterol to high-density lipoprotein (HDL) particles (5, 42). HDL is believed to play a protective role in atherosclerosis but raising the HDL level by inhibiting cholesteryl ester transfer protein did not reduce cardiovascular risk in several studies (20, 43). The major cholesterol uptake mechanism involves CD36 and SR-A (44). 75%–90% of modified lipoproteins are transported through CD36 and SR-A (Figure 1). Endoplasmic reticulum acetyl-CoA acetyltransferases (ACAT) generate cholesterol ester (45). HSPs are recognized as lipid metabolism regulators because they affect the expression of genes involved in cholesterol absorption (such as CD36 and LOX1) (46, 47) and efflux (ABCA1, ABCG1) (48, 49). Furthermore, HSPs have the capacity to control inflammatory pathways, produce cytokines, and aid in the death of foam cells (50).

HSP72 isoforms may function by themselves or in concert with HSP90, where they are crucial in the substrate recruitment (51). In addition to acting as chaperones, HSP72 isoforms promote cell survival by inhibiting apoptosis at several processes across intrinsic and extrinsic cell death pathways (52, 53). Furthermore, HSP72 induces a strong transcriptional re-programming, including the overexpression of critical targets of liver X receptors (LXR), master regulators of systemic cholesterol elimination (54, 55). As a result of HSP72's interaction with the macrophage LXRα promoter, the expression of LXRα and LXRα’s target genes, including ABCA1 and ABCG1, are increased and, consequently, their protein abundances are enhanced.

On the other hand, HSP72 increases the amount of lipids accumulated in arteries and contributes to the development of atherosclerotic lesions. In mice lacking ApoE, HSP72 accelerates the development of atherosclerosis by inhibiting the production of ABCA1 and ABCG1 in peritoneal macrophages and the aorta through the JNK/Elk-1 pathway (12). Moreover, a positive correlation between the expression of HSP72 and the stability of advanced human atherosclerotic plaques has been shown. The formation of atherosclerotic lesions is also linked to the inhibition of the expression and the activity of Sirtuin 1 (SIRT1), which, in turn, underlies both the expression of HSF1 and HSPs and the ability of HSF1 to transcribe genes (56).

HSP27 has been proposed as a biomarker and therapeutic target in atherosclerosis. Through the PI3K/PKC/Sp1 pathway, HSP27 stimulates ABCA1 expression and cholesterol efflux in THP-1 macrophage-derived foam cells (48). Through a mechanism involving NF-kB signaling, HSP27 suppresses SR-A expression, hence reducing foam cell production and atherogenesis. HSP27 is atherosclerosis protective when it is overexpressed and its level in circulation is elevated, this HSP27-mediated protection *in vivo* requires SR-A as a cofactor (50).

Mouse Hsp25, the ortholog of human HSP27, has been administered as a vaccine to inhibit atherogenesis in mice, it upregulates low-density lipoprotein receptor (LDLR) expression, reduces proprotein convertase subtilisin/kexin type 9 (PCSK9) levels, and suppresses inflammation (57). Cholesterol levels may be also reduced by Hsp25 immunotherapy, Hsp25 reduces plaque cholesterol by 30% and prevents experimental atherosclerosis in mice, which requires GM-CSF-regulated ABCA1 and ABCG1 expression (49).

## Controversial roles of HSPs in atherosclerosis

The role of HSPs in atherosclerosis is contentious with some studies suggesting a protective role by preventing protein accumulation and inhibiting inflammation while others suggesting a disease progression promoting role by increasing cell migration and proliferation and activating inflammatory pathways (58). A study shows that healthy people have lower levels of extracellular HSP72 than patients with cerebrovascular atherosclerosis, indicating that extracellular HSP72 may be protective against the development of this illness (59). However, high levels of extracellular HSP72 are detected in individuals with severe arterial calcification and are correlated with an increased risk for the development of arterial calcification (60). It is reported that the anti-inflammatory pathway is suppressed in atherosclerosis (61). Differences in the types of HSPs studied and experimental models used may contribute to these conflicting results, further research is needed to clarify the roles of HSPs in atherosclerosis.

HSPs have different effects on atherosclerosis depending on the stage of aortic atherosclerosis (11). In early stages of atherosclerosis, induction of HSPs protects against aortic atherosclerosis *via* lowering adhesion molecule expression. On the other hand, induction of HSPs promotes aortic atherosclerosis by increasing the production of inflammatory cytokines and activating macrophage activity as atherosclerosis progresses (Figure 1). Thus, HSPs induction and inhibition should be investigated for the prevention and treatment of atherosclerosis, respectively (11).

## Heat shock factor 1 (HSF1) is essential for PSR

In order to better understand HSPs' function in atherosclerosis, it is important to understand their upstream regulation. Among multiple signaling pathways that regulate HSPs expression in atherosclerosis, the HSF1 pathway is a dominant mechanism in response to stresses (34). During physiological and pathological processes, inflammatory cytokines and reactive oxygen species may also increase HSF1 activation (62). In atherosclerotic lesions, HSF1 is activated and cytokine stimulation causes HSF1 nuclear translocation, DNA binding, and to increase HSP72 expression (39). Therefore, HSF1 could be a novel target on atherosclerosis.

There are nine HSF family members in mammalians, including HSF1, 2, 3, 4, 5, X1, X2, Y1, and Y2. These nine members have a variety of distinct and overlapping roles and characteristics (34, 63). The N-terminus of HSF proteins contains a helix-turn-helix DNA-binding domain (DBD). However, this helix-turn-helix domain of HSF1 does not interact directly with DNA; rather, it stabilizes the HSF1 trimer that is bound on DNA (64, 65). Under heat shock, inactive monomeric HSF proteins form homotrimer, translocate to the nucleus, interact with heat-shock elements (HSEs) in the genome, and initiate the transcription of their target genes. HSEs are comprised of several inverted repetitions of the pentanucleotide motif nGAAn, different HSF family members have preferences for different HSEs, resulting in the transcription of a wide variety of target genes, including those encoding HSPs (Figure 1). Upon the resolution of the stress or after prolonged stress, HSF proteins revert to a monomeric state and their transcriptional activities decrease (34, 66).

**Figure 2:**
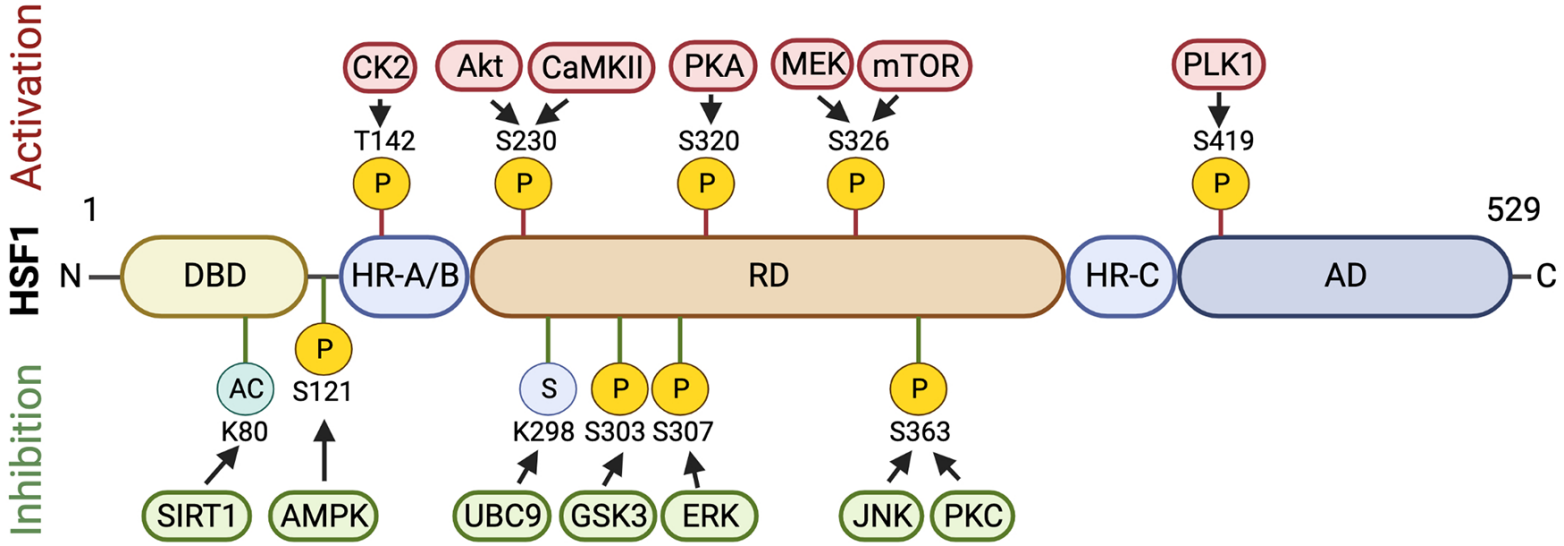
HSF1 regulatory domains and post-translational modifications. HSF1 is composed of several functional domains, including a DNA-binding domain (DBD), a regulatory domain (RD), a heptad repeat (HR), and a transactivating domain (AD). Specific sites for serine/threonine phosphorylation (P), acetylation (AC), and SUMOylation (S) that activate or inhibit HSF1's activity are shown. These post-translational modifications are mediated by a variety of kinases, de-acetylases, and SUMOylases. AMPK, AMP-activated protein kinase, Akt, protein kinase B; CaMKII, calcium/calmodulin-dependent protein kinase II; CK2, casein kinase 2; ERK, extracellular signal-regulated kinase; GSK3, glycogen synthase kinase 3; JNK, c-Jun N-terminal kinase; mTOR, mammalian target of rapamycin; MEK, mitogen-activated protein kinase; PKA, protein kinase A; PKC, protein kinase C; PLK1, polo-like kinase 1; SIRT1, sirtuin 1; UBC9, ubiquitin-conjugating enzyme E2I. Images were created with BioRender.com.

HSF1 controls the chaperon protein production in order to change the misfolded proteins when cells are exposed to a stressor, such as increased temperature (67, 68). HSF1 is the most important transcription factor for the transcription of HSP genes in eukaryotes (69, 70). For example, fibroblasts from mice lacking HSF1 lack stress-induced transcription of HSP genes, indicating that HSF1 is sufficient for HSP production (71). Thus, HSF1 is the major factor determining cellular response to proteotoxic stimuli. HSF1 comprises several functional domains (Figure 2). The oligomerization domain of HSF1 is composed of leucine-zipper-like heptad repeat region A (HR-A) and HR-B, which together establish an all-parallel structure to control HSF1 trimerization. The HR-C region is believed to maintain HSF1 at the inactive state by inhibiting HSF1 trimerization (72, 73). The activation domain (AD) of HSF1 promotes the transcriptional activation of HSF1's target genes and controls HSF1's activity (74, 75). The regulatory domain (RD), situated between HR-A/B and HR-C regions, is important for inhibiting HSF1 function in the absence of stress. In addition, the RD of HSF1 is believed to contain a heat shock sensor and functions as the target for several induced post-translational modifications (76, 77).

Protein-protein interactions and post-translational modifications (PTMs), such as phosphorylation, SUMOylation, and acetylation, influence the activation of HSF1 (62, 78). Figure 2 illustrates PTMs on HSF1 that modulate HSF1's activity and enzymes that are reported to be responsible for these PTMs. Acetylation of HSF1 inhibits HSF1 DNA binding. After exposure to heat, HSF1 trimers bind to HSE and are phosphorylated and SUMOylated at many sites to control its activity. For example, at least 12 phosphorylation sites of HSF1 have been identified, some of which activate whereas others inhibit HSF1 (34, 69, 79). Among these, phosphorylation of HSF1 at S326 in response to stress stimulates HSF1 activation whereas phosphorylation of HSF1 at S303/307 inhibits HSF1's activity in the absence of stress (80, 81). In addition, in HEK293 cells, metformin treatment promotes the phosphorylation of HSF1 at S121 and decreases HSF1's DNA-binding activity (82). Accordingly, ectopically expressed HSF1 S121A mutant is insensitive to the metformin-induced decrease in DNA-binding activity in cells with their endogenous wild type HSF1 being knockdown (82). HSF1 phosphorylation therefore may play a role in atherosclerosis. For example, HSF1 phosphorylation at Ser326 activates HSF1 to upregulate HSPs expressions, which can protect against atherosclerosis. It is possible that different HSF1 phosphorylations may have different effects on HSPs expressions and atherosclerosis. Moreover, kinases and signaling pathways that regulate HSF1 phosphorylation in atherosclerosis have not been fully characterized. Further research is needed to elucidate the precise role of HSF1 phosphorylation in atherosclerosis and mechanisms by which HSF1 phosphorylation affects atherosclerosis and to determine whether targeting HSF1 phosphorylation could be a viable therapeutic strategy for the prevention or treatment of this disease.

## HSF1 mediates lipid homeostasis and atherosclerosis

AMP-activated protein kinase (AMPK) is the primary regulator of cellular and body energy homeostasis, including glucose and lipid metabolism (83, 84). Animal model studies show that increasing oxidative metabolism improves insulin resistance. For example, activation of AMPK or SIRT1 increases fatty acid oxidation, which decreases lipid esterification and improves insulin resistance (85, 86). Furthermore, AMPK is an important sensor of cellular energy status, its activity is dramatically reduced in insulin resistance whereas activating AMPK promotes insulin sensitivity (87). Furthermore, Cushing's syndrome is defined by reduced activation of AMPK in conjunction with insulin resistance in adipose tissue (88). AMPK activity is reduced in the adipose tissue of highly obese people (89). AMPK-regulated lipid metabolism is particularly crucial in the control of cardiovascular disorders such as atherosclerosis and endothelial-related vascular diseases (90).

In addition to its transcriptional function, HSF1 also non-transcriptionally regulates lipogenesis and cholesterol synthesis. HSF1 stimulates lipid synthesis through many signaling pathways, including suppressing AMPK to induce lipogenesis (8). Knockdown HSF1 reduced lipid droplet (8). In human melanoma cells, inducible HSF1 knockdown results in increased sterol regulatory element-binding protein 1c (SREBP1c) Ser372 phosphorylation, decreased levels of acetyl-CoA carboxylase (*ACC1*), fatty acid synthase (*FASN*), *LDLR*, and 3-Hydroxy-3-Methylglutaryl-CoA Reductase (*HMGCR*) mRNAs, and reduced SREBP1's nuclear translocation and binding to genomic DNA (8). Primarily *via* inhibiting the AMPK activity, HSF1 is responsible for the transcriptional activation of SREBP1 (8, 91, 92). Interestingly, HSF1 peptides reduce AMPK's function by preventing AMPK binding to ATP and AMP, promoting phosphatase engagement with AMPK, and eventually leading to AMPK conformational shift, which limits AMPK's activity (8). Thus, HSF1 reduces AMPK's activation by directly interacting with it (Figure 1).

HSF1 also mediates mevalonate and cholesterol biosynthesis. Inhibition of HSF1 by using its inhibitor KRIBB11 or shRNA reduces H-RasV12-induced cholesterol production (91). How HSF1 affects intracellular cholesterol homeostasis is unknown. However, because HSF1 deficiency-induced cellular cholesterol reduction is counteracted by AMPK knockdown, this regulation appears to involve AMPK (8). Statins stabilize HMGCR and SREBP2-mediated cholesterol production, reducing their heart disease therapy effectiveness. These findings suggest that suppressing HSF1 may enhance statin-based cholesterol reduction (91). Inhibiting HSF1 by KRIBB11 significantly reduces the expression of lipogenic enzymes, including ACC and FAS, and increases β-oxidation-associated enzyme palmitoyltransterase-1 (93). These findings indicate that HSF1 plays a crucial role in mediating intracellular lipid metabolism. The HSF1-AMPK signaling may be a promising therapeutic target for lipid dysregulation.

HSF1 has been reported to have a protective role in endothelial cells, HSF1 activation promotes the expression of HSPs and protects against oxidative stress-induced vascular injury and induces angiogenesis (94, 95). HSF1 activation induces the expression of the endothelial nitric oxide synthase to maintain the endothelial function *via* the nitric oxide (NO) production (94). Besides, low level of NO induces HSP70 expression in vascular SMCs through the induction of HSF1 activity (96). HSF1-induced HSPs expression also protects vascular SMCs from calcification (97). In addition, it has been reported that HSF1 suppresses the lung injury *via* suppressing macrophage infiltration (98). Nevertheless, the evidence of HSF1 regulation in macrophage-foam cell in atherosclerosis is limited.

HSF1 might be a nice marker in obesity-related disorders, including atherosclerosis (99). HSF1 is reported to be activated in atherosclerosis (39). Furthermore, HSF1 deficiency increases the expression of cholesterol 7-hydroxylase (CYP7A1) and multidrug transporter (MDR1) genes, hence reducing atherosclerosis (100). HSF1 deficiency also significantly represses PPAR-γ2, a nuclear receptor that controls the vast majority of genes involved in the lipid metabolism (100). HSF1 inhibition improves the lipidemic profile through enhancing cholesterol clearance and lowers atherosclerotic lesion load, making it a plausible target in atherosclerosis management. These results indicate that HSF1 has pleotropic roles in atherosclerosis. Nonetheless, the detailed mechanism for how HSF1 regulates atherosclerosis through mediating lipid homeostasis requires further investigated.

## Discussion

This review discusses an intimate relationship between atherosclerosis and PSR, focusing on HSF1 and HSPs in atherosclerotic lipid homeostasis. HSF1 and HSPs serve crucial roles in the quality control of proteins, particularly in regulating protein misfolding, aggregation, and refolding. Atherosclerosis is caused by imbalanced inflammation, lipid composition, as well as lipid uptake and efflux. These stresses induce the activation of multiple responses, including HSPs expression, at various stages of atherosclerotic pathogenic progression. HSF1 and its functionally associated kinases, such as AMPK, control both proteotoxic stress and lipid metabolism, albeit *via* distinct mechanisms. Notably, HSPs expressions at different stages of atherosclerotic lesions have opposing effects in the progression of atherosclerosis. Therefore, therapeutic approaches targeting HSPs at various stages of atherosclerosis should be administered with caution. Finally, we infer that the mechanism of HSF1-mediated lipid metabolism may present novel therapeutic targets for cardiovascular disorders linked with atherosclerosis and that the activation of HSF1 and HSPs may serve as diagnostic indicators for atherosclerosis.
